# Polysaccharide Hydrogels for the Protection of Dairy-Related Microorganisms in Adverse Environmental Conditions

**DOI:** 10.3390/molecules26247484

**Published:** 2021-12-10

**Authors:** Ilja Gasan Osojnik Črnivec, Tigran Neresyan, Yuliana Gatina, Vid Kolmanič Bučar, Mihaela Skrt, Iztok Dogša, Bojana Bogovič Matijašić, Irina Kulikova, Aleksei Lodygin, Nataša Poklar Ulrih

**Affiliations:** 1 Biotechnical Faculty, University of Ljubljana, 101 Jamnikarjeva, 1000 Ljubljana, Slovenia; gasan.osojnik@bf.uni-lj.si (I.G.O.Č.); v.kolbuc@gmail.com (V.K.B.); mihaela.skrt@bf.uni-lj.si (M.S.); iztok.dogsa@bf.uni-lj.si (I.D.); bojana.bogovicmatijasic@bf.uni-lj.si (B.B.M.); 2 Food Engineering and Biotechnology Faculty, North-Caucasus Federal University, 1 Pushkin Street, 355017 Stavropol, Russia; tigran_nersesyan@mail.ru (T.N.); yuliana.gatina@yandex.ru (Y.G.); kik-st@yandex.ru (I.K.); allodygin@yandex.ru (A.L.); 3 The Centre of Excellence for Integrated Approaches in Chemistry and Biology of Proteins, 39 Jamova, 1000 Ljubljana, Slovenia

**Keywords:** encapsulation, polysaccharides, hydrogels, probiotics, dairy products, lactic acid bacteria, lactose-fermenting yeasts

## Abstract

Adverse environmental conditions are severely limiting the use of microorganisms in food systems, such as probiotic delivery, where low pH causes a rapid decrease in the survival of ingested bacteria, and mixed-culture fermentation, where stepwise changes and/or metabolites of individual microbial groups can hinder overall growth and production. In our study, model probiotic lactic acid bacteria (*L. plantarum* ATCC 8014, *L. rhamnosus* GG) and yeasts native to dairy mixed cultures (*K. marxianus* ZIM 1868) were entrapped in an optimized (cell, alginate and hardening solution concentration, electrostatic working parameters) Ca-alginate system. Encapsulated cultures were examined for short-term survival in the absence of nutrients (lactic acid bacteria) and long-term performance in acidified conditions (yeasts). In particular, the use of encapsulated yeasts in these conditions has not been previously examined. Electrostatic manufacturing allowed for the preparation of well-defined alginate microbeads (180–260 µm diameter), high cell-entrapment (95%) and viability (90%), and uniform distribution of the encapsulated cells throughout the hydrogel matrix. The entrapped *L. plantarum* maintained improved viabilities during 180 min at pH 2.0 (19% higher when compared to the free culture), whereas, *L. rhamnosus* appeared to be less robust. The encapsulated *K. marxianus* exhibited double product yields in lactose- and lactic acid-modified MRS growth media (compared to an unfavorable growth environment for freely suspended cells). Even within a conventional encapsulation system, the pH responsive features of alginate provided superior protection and production of encapsulated yeasts, allowing several applications in lacto-fermented or acidified growth environments, further options for process optimization, and novel carrier design strategies based on inhibitor charge expulsion.

## 1. Introduction

Microencapsulation typically involves coating or entrapping a core component within a polymer to generate microspheres, microcapsules, or micro aggregates in the range of 1–1000 μm [1]. In recent decades, this technology has been widely applied to microbial cell immobilization, offering high cell-loading capacities and physical isolation from the external environment [2,3].

In food systems, efficient cell-entrapment techniques are particularly in demand for probiotics and fermentation/starter cultures. For ingestible probiotics, the encapsulation carrier provides protection during storage and digestion, as well as in more advanced applications, acting as a delivery system to pass through and reach targeted parts of the digestive tract. This protective effect is important, as the efficiency of ingested probiotics depends on the dose of viable cells that reach their intended destination in the gut [4]. In the case of encapsulation of starter cultures used in various fermentation processes, the goals are to shorten the period of adaptation in the fermentation broth, achieve high cell density within the carrier, improve the kinetics of the process, reduce microbial contamination, and allow for easier separation and reuse of biomass for the next batch [3,5].

Several lactic acid bacteria, which are isolated from food or are of human origin, exhibit probiotic attributes and are typically the most common probiotics in foods, are often used as key ingredients in food supplements or functional foods. Their health-promoting effects are widely known and include stabilization of gut microbiota, improvement of intestinal health, enhancement of immune responses, reduction of serum cholesterol levels, and increased nutritional value of foods. Among lactobacilli, commonly used probiotic products include *Lactobacillus* spp. (i.e., *L. acidophilus*, *delbrueckii*, *johnsonii*), *Lacticaseibacillus* spp. (i.e., *L. casei*, *paracasei*, *rhamnosus*), *Levilactobacillus brevis*, *Lactiplantibacillus plantarum* and *Limosilactobacillus fermentum* [6,7,8,9,10,11].

Due to the abundance of natural biopolymers with diverse surface and colloidal properties, several approaches have been applied for encapsulation of microorganisms in food. For probiotics based on lactic acid bacteria, preparation of hydrogels and emulsions are most common. These encapsulation systems are frequently layered and incorporate prebiotics, and are tested in a nutrient-rich environment. Ding and Shah [12] performed an extensive study of hydrogel carriers for ten strains of dairy-derived probiotics (including *L. plantarum* Lpc-37, *L. rhamnosus* r-32, and *L. rhamnosus* HOWARU) and reported alginate, xanthan gum, and carrageenan gum as most effective in protecting probiotic cells from harsh environmental conditions. Galacturonan-rich pectin and other mixed polysaccharides, such as alginate-gum arabic, have also been found to provide good structural properties for the delivery of microbes [13,14]. Creating multiple zones within the encapsulation system, with different chemical properties such as chitosan-coated alginate hydrogel beads [15,16] or double *w/o/w* emulsions [17], can maintain high survival rates. Multiple coatings bring additional features and can achieve even higher levels of protection [18,19].

However, layering requires additional ingredients and process steps, as well as longer preparation times. Further probiotics encapsulation approaches are based on gelling prebiotic polysaccharides or the inclusion of prebiotics in hydrogels, which improves the viability of cells and affect the structural integrity of hydrogels [20,21,22]. The survival of lactic acid bacteria is enhanced by the presence of metabolizable sugars. In this respect, it must also be considered that in many cases nutrients and cells are simultaneously loaded into the core [13,14,23], or the cells are tested for their acid tolerance in a nutrient-rich environment [12,15,24,25]. However, during encapsulation, as well as in the gastrointestinal tract, the availability of nutrients is often decreased. The described delivery systems provide good protection for encapsulated probiotics, but with a tradeoff of an increasing degree of complexity that ultimately leads to lower feasibility.

Furthermore, several fermented foods, such as mixed-culture dairy products, sour beer, and sourdough bread, are the products of mixed fermentation of lactic acid bacteria and yeasts. Such mixed fermentation is often a two- or multiple-stage process, particularly in dairy products such as ayran and kefir, where lactic acid bacteria such as *Lentilactobacillus kefiri, L. paracasei*, *L acidophilus, L. delbrueckii* spp. *bulgaricus*, *L. plantarum*, and *Lactobacillus kefiranofaciens* are often accompanied by yeasts such as *Saccharomyces* spp. (e.g., *S. cerevisiae, S. unisporus*)*, Candida* spp. (e.g., *C. kefyr*), and *Kluyveromyces* spp. (e.g., *K. marxianus).* Other microbial genera associated with these milk products are *Streptococcus* (particularly *S. thermophilus*, dominating the ayran microbiota with *L. delbrueckii* subspp. *bulgaricus*), *Acetobacter, Bifidobacterium,* *Lactoccus*, *Leuconostoc*, *Bretanomyces*, *Kazachastania, Naumovozyma, Pseudomonas, Torulaspora*, and others [26,27,28]. These fermented products can be considered as functional foods, as they have a number of beneficial dietary properties, including alleviation of lactose intolerance, reduction of cholesterol levels, boosting of the immune system, antimicrobial and anti-inflammatory effects, and propagation and proliferation of intestinal probiotic microorganisms [29,30]. Mixed cultures are employed to markedly decrease sugar content, improve organoleptic properties, and prolong the shelf life of the product.

The primary fermentation in the production of kefir or ayran is performed by lactic acid bacteria at mesophilic or thermophilic conditions, whereas lactose-fermenting yeasts are involved in later ripening stages of fermentation, as well as during storage. Therefore, during primary fermentation, the environmental conditions (titratable acidity, pH, and redox potential) are unfavorable for yeast growth. During subsequent ripening and storage, the yeasts become the predominant microorganisms that enable the product to obtain a light yeasty taste and a specific refreshing odor [31,32]. The shelf life of these products is limited by yeast growth and consequential gas formation. Prolonged shelf life can be achieved with the addition of salt [33], subject to obvious dietary constraints. Moreover, specific yeast strains can be also used to achieve low gas and alcohol production; however, this often results in decreased organoleptic properties in the final products, in particular with respect to overall freshness and taste [34]. Due to the dual nature of the process and the composition of modern mixed-culture dairy starters, there is an opportunity to improve these processes via the encapsulation of yeasts.

*Kluyveromyces* spp. are a common companion to lactic acid bacteria, frequently dominating fungal populations of milk products that are produced through mixed-culture fermentation [27]. These native yeasts have also been used recently for the production of bioethanol, enzymes, and other high-value chemicals from lactose [35,36], as well as for the introduction of novel organoleptic profiles to beer [37,38]. To date, there have been few encapsulation approaches for food applications, mostly focused on *K. lactis* and *K. marxianus*. Most encapsulation and immobilization approaches have been studied for probiotic delivery or for the biotechnological production of lactose, bioethanol, or other biochemicals [35,39,40,41]. Examples of insoluble and/or hydrogel carriers include the entrapment of *K. lactis* in glutaraldehyde cross-linked gelatin, where 10% encapsulation efficiency and 95% to 50% viability in packed bed fermentation and simulated gastrointestinal passage was reported, respectively [35]. In a separate study, graphene oxide was introduced into this system as a reinforcement agent [36]. The encapsulation of *K. marxianus* has been reported in derivatised chitosan beads and studied for their joint antimicrobial activity against pathogenic bacteria as an alternative to antibiotics in animal feed. Here, the entrapped microbes exhibited no decrease in viability during the simulated gastrointestinal passage [42]. Furthermore, another study examined *K. marxianus* as a starter culture for baking and cheese-ripening. After long-term storage at 4 °C, the cultures that were absorbed on micro/nanocellulose fibers exhibited high activity and 50–100% longer lactose and whey fermentation times, compared with freshly dried encapsulates [43]. Foamed alginate carriers were also used for the immobilization of *K. marxianus* grown on apple/chokeberry and apple/cranberry pomaces, providing cell growth comparable to free cultures and novel metabolic profiles of aromatic compounds in encapsulated cells [44]. Various food applications are also focused on whole-cell encapsulation of *Kluyveromyces* spp. that focus on preserving a high enzymatic activity of the encapsulate, and not on overall cell survival [39,45,46].

Therefore, in a system consisting of several layers, probiotics, prebiotics, and available nutrients, the roles of individual ingredients and the inherent properties of the entrapped cultures cannot be clearly distinguished, e.g., at the low pH (1.5–5.0) that is expected in the stomach [47]. Complex carrier systems can strongly affect the cost of encapsulation, obscure the properties and behavior of individual carrier components, and limit application to food systems via approved food additives. In the EU database on Food Additives (Regulations (EC) 1333/2008, (EU) 2018/1497, (EU) 2015/647, (EU) 2015/1832, (EU) 1129/2011), food additives from the alginate family (E400-E404) are approved for a wide variety of food and drinks, such as flavored and alcoholic drinks, confectioneries, dried fruit and vegetables, meat preparations, food supplements, and even processed foods and baby foods for infants and young children. Admixing alginate with other polysaccharide components that may be similar in chemical composition, such as pectins (E 440), already changes possible applications in food products. On the other hand, other popular approaches, such as coacervation or layering with chitosan, require that all applications beyond food supplements must be authorized under the Novel Food Regulation (EU) 2015/2283.

Therefore, to study the protective role of microencapsulation on microbial survival and activity, model probiotic lactic acid bacteria and kefir-derived native yeast were tested in a conventional Ca-alginate system. Furthermore, there is limited information on maintaining the activity of native yeasts when metabolism is slowed down by acid inhibition, namely during multi-stage mixed-culture milk fermentation. To our knowledge, the design and examination of a system for subsequent introduction of yeasts within a food matrix, where lactic acid fermentation has already occurred, has not been previously attempted.

The cell, alginate and hardening solution concentration, and other working parameters were optimized, and the obtained microbeads were exposed to unfavorable growth conditions. The short-term survival (180 min, 37 °C, in de Man, Rogosa and Sharpe broth (MRS)) of *L. plantarum* ATCC 8014 and *L. rhamnosus* GG bacteria was examined at low pH (2.0) that is expected in the stomach. Furthermore, the long-term performance (48 h, 28 °C, in MRS where glucose was replaced by lactose (MRSL) or in MRSL acidified with lactic acid (MRSLL)) of *K. marxianus* ZIM 1868 yeasts was examined in the presence of a lactic acid inhibitor (at pH 4.2) at levels expected during the second stage of mixed-culture fermentation. A combination of standard plating methods, confocal laser scanning microscopy, substrate uptake/product formation, and growth media composition modification was applied to understand the exhibited survival profiles, growth dynamics, and microbial activity.

## 2. Results and Discussion

When alginate microbeads were prepared with microbial cells the particle size increased, in contrast to pure alginate hydrogels (180 ± 20 µm). The beads containing lactic acid bacteria in 0.9% NaCl were larger (260 ± 30 µm) compared to beads containing yeasts in MRSL (190 ± 20 µm). In both cases, 95% cell-entrapment in alginate and a decrease in viability of up to 10% was estimated via differential staining and standard plating methods. When these hydrogels were introduced into solutions with pH 2.0, i.e., at values below the pK_a_ (3.2 and 4.0) of alginate, hydrogel swelling was suppressed [48], resulting in 30% smaller microbeads. Furthermore, to confirm the suitability of the applied laboratory techniques, aseptic conditions during encapsulation and the extent of bead dissolution were also examined by microscopy for empty and microorganism-containing beads (see the Supplementary Materials, Figures S3 and S4).

**Figure 1:**
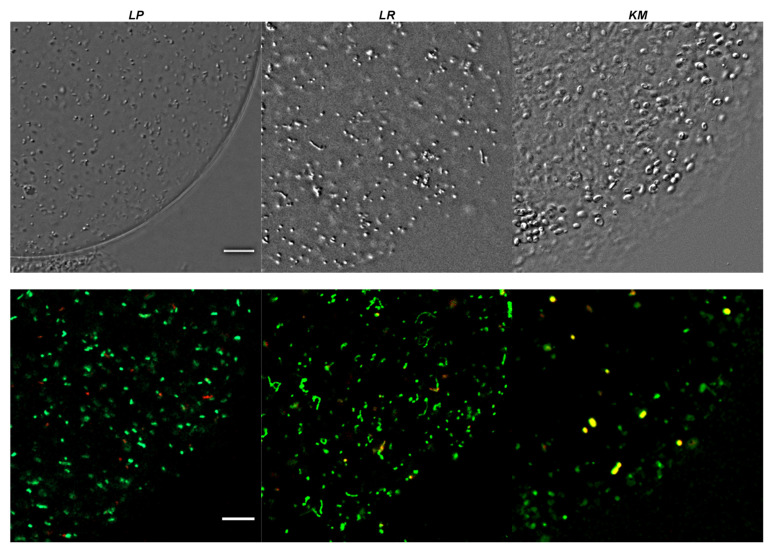
Survival and spatial distribution of (**LP**) *L. plantarum*, (**LR**) *L. rhamnosus* and (**KM**) *K. marxianus* within microbeads after encapsulation. Top row shows differential interference contrast micrographs and bottom row (2) shows cells stained by live (SYTO 9 dye)/dead (propidium iodide dye) kit. The red and yellow cells are dead cells, whereas only green cells are alive. Scale bar corresponds to 20 µm.

In all tested cases, the results showed formation of well-defined spherical hydrogel particles and good survival of lactic acid bacteria and yeasts throughout the encapsulation process, as observed via confocal laser scanning microscopy (Figure 1). These observations also showed that encapsulated cultures were set throughout the cross-linked hydrogel and were distributed relatively uniformly from the center to the outer boundary of the microbeads.

### 2.1. Short-Term Survival of Encapsulated Lactic Acid Bacteria

**Figure 2:**
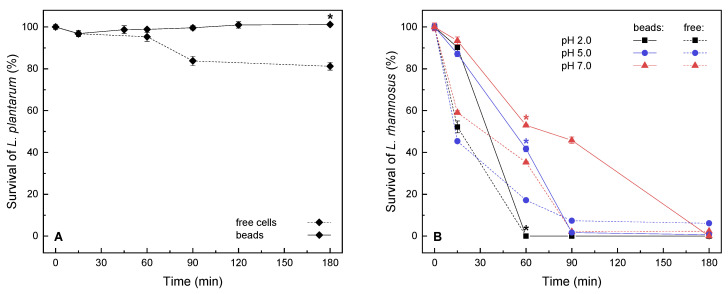
Short-term survival for encapsulated and freely suspended bacterial cultures of (**A**) *Lactiplantibacillus plantarum* at pH 2.0 and (**B**) *Lacticaseibacillus rhamnosus* GG at pH 2.0–7.0. Survival shown as proportion of live cells at the time of sampling vs. the start of incubation. Data are means ± standard deviation (*n* = 3). Free cultures were taken as the reference; dissimilar curves (*f*_2_ < 50) are marked with an asterisk (*).

Tolerances to low environmental pH (2.0) for free and encapsulated probiotics (*L. plantarum*, *L. rhamnosus*) were examined in a relatively short-term window (up to 180 min) as a means of obtaining basic information on probiotic survival during gastric passage. With or without encapsulation in 2% alginate beads, the bacterial cultures were washed, exposed to the hardening solution, and subsequently transferred to the buffer (Figure 2). During encapsulation and exposure to the hardening solution, the decrease in viability (percentage of live/total cells) was more evident for freely suspended cells (91% ± 1%, 52% ± 7%) than for cells in alginate beads (92% ± 1%; 90% ± 3%) for the respective cultures of *L. plantarum* and *L. rhamnosus,* indicating that the latter strain might be less robust. During the following incubation, the integrity and the shape of alginate microbeads stayed intact in all observed samples.

The survival of the model acid-tolerant *L. plantarum* probiotic [49] was examined at low pH and under starvation conditions (Figure 2A), as the presence of certain growth medium components, such as carbohydrates, can also promote survival [25]. During the three-hour exposure to these conditions, the entrapped *L. plantarum* maintained viability similar to initial levels, whereas the number of dead cells within the freely suspended culture started to increase after the first hour. In three hours, overall survival was enhanced for 19% by encapsulation, as compared with suspended bacteria. These results are similar to the 87% survival reported in simulated gastric juice (120 min at pH 3.0) for *L. plantarum* MTCC 021 encapsulated in resistant starch microbeads; however, the survival of free cells was much lower (13%) [9]. Furthermore, other sources also report a high variability in the survival of unencapsulated cultures under environmental stress for different strains of *L. plantarum* [10,11].

To gain more information on possible applications for probiotics, encapsulation of *L. rhamnosus* GG was investigated. For *L. rhamnosus*, cell death was drastically delayed in microbeads at pH 2.0 (Figure 2B) but reached similarly low values for both suspended and entrapped cells after three hours of exposure in an acidic and hypotonic environment. As similar results have been described in the literature, indicating that the acid tolerance of *L. rhamnosus* is relatively high but might be lower than that of *L. plantarum* [50], the survival at pH 5.0 and 7.0 was also examined (Figure 2B). For different pH values, the protective effect of hydrogel entrapment was most evident at different time points during the experiment, i.e., for pH 2.0 at the initial stages of exposure (similar protection for all pH values up to 40%); for pH 5.0 after 60 min of exposure (improved for ~24%); and for pH 7.0 after 90 min (improved for 44%). The overall highest survival of *L. rhamnosus* was achieved in alginate microbeads at pH 7.0. Furthermore, at the first time of sampling, an initial discrepancy in survival could be observed among the freely suspended and entrapped *L. rhamnosus*. This might suggest a prolonged response to the hardening solution, as a control exposure was also performed for free cells; this initial stress factor could explain the lower survival exhibited even at higher pH values (Figure 2B). At this initial time, a steep (48%) decrease in survival was exhibited in free cells, whereas entrapped cells exhibited less than a 10% decrease in survival during gelation. To a lesser extent, the initial decrease in survival could be seen for *L. plantarum*, with a negligible difference in survival between the free and encapsulated cells (Figure 2A).

The results point to varied inherent tolerances to isotonic starvation when *L. plantarum* and *L. rhamnosus* are compared. Although the tolerance of low pH by *L. plantarum* and *L. rhamnosus* has not yet been directly compared in the available literature, our own previous observations [51] and some other studies showed lower survival of *L. rhamnosus* (GG or other strains) as compared to other acid-tolerant lactic acid bacteria at low pH, during short-term starvation and/or exposure to bile salts [25,52]. Moreover, a similar albeit slightly extended survival profile was reported for *L. rhamnosus* in alginate microbeads in a nutrient-rich environment, i.e., in MRS adjusted to pH 2.0 [15]. Furthermore, for both studied strains, the protective capacity of encapsulation was demonstrated even in inadequate growth conditions in our study, and further outlines for optimization could be taken from the literature [25], such as the inclusion of metabolizable components and/or prebiotics in the encapsulation matrix, particularly for applications where long-term growth and activity are required.

### 2.2. Fermentation Activity in Encapsulated Yeasts

In addition to their frequent probiotic role, lactic acid bacteria are traditionally used in the production of fermented foods, quite often acting in mixed cultures with yeasts. In such microbial communities, lactic acid bacteria typically provide transformation of soluble sugars to carboxylic acids (primarily to lactate) in the first fermentation step. During this process, the conditions are relatively unfavorable for yeast growth, limiting their activity for the uptake of remaining nutrients in secondary fermentation. In the industrial production of fermented milk products, kefir grains and native complex ayran cultures are no longer used; instead, a powdered preparation of bacteria and yeast is used to allow the flavor to be kept constant. Commercial starters are prepared by combining three to four strains of known bacteria and yeasts, making it possible to consider separate encapsulation of individual strains.

**Figure 3:**
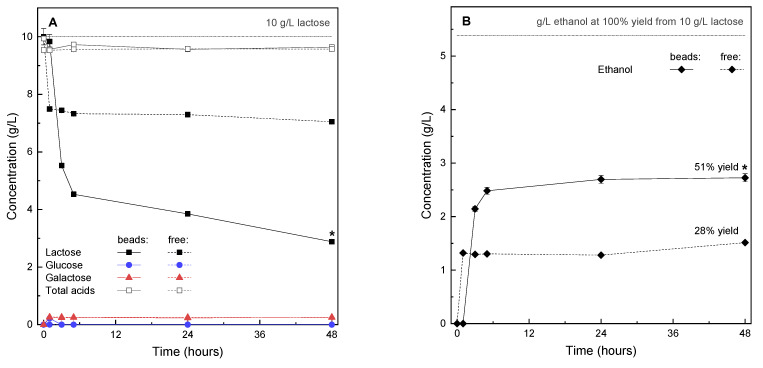
Incubation of *Kluyveromyces marxianus* ZIM 1868 in MRSLL (48 h, 28 °C) monitored by (**A**) substrate consumption and (**B**) ethanol production. Data are means ± standard deviation (*n* = 3). Free cultures were taken as the reference; dissimilar curves (*f*_2_ < 50) are marked with an asterisk (*).

To examine how encapsulation could provide novel possibilities for such a transformation of milk products, as well as bring novel aromatic profiles to fermented drinks, we considered the addition of encapsulated yeasts to the partially transformed food matrix after lactose fermentation to lactic acid had concluded. For this process, *K. marxianus*, which is used in kefir and sour beer production, was cultured and studied in MRSLL, which provided a lactose- and lactic acid-rich growth environment (Figure 3). Surprisingly, the encapsulated yeasts exhibited significantly higher amounts of lactose conversion and ethanol production, amounting to double product yields (Figure 3B) in comparison to freely suspended cells. Furthermore, in all cases, ethanol production peaked in the first hours of fermentation, and the inhibition of production could already be seen at the level of lactose hydrolysis, resulting in fast cellular uptake and insignificant residual monosaccharide concentrations in the culture broth (Figure 3A).

Several studies show that various *K. marxianus* strains are able to grow in the presence of relatively high amounts of lactic acid [53,54,55,56]. However, the increased performance in encapsulates in comparison to free yeast performance, as shown in Figure 3, might suggest that this particular strain is less tolerant to lactic acid. Furthermore, it must be taken in account that the strains discussed in the literature were obtained for environments with high-acidity (silage or sauerkraut leachates) or high-ethanol content (distilleries etc.), while the strain in our study was isolated from kefir grains in which the levels of lactic acid and end ethanol are relatively low. Moreover, when *K. marxianus* was considered as an establishing model in bioethanol production from whey, no limiting effects of lactic acid were reported at 0 to 15 g/L, and lower ethanol productivity was observed at 30 g/L, providing a benchmark at >15g/L [55], which roughly corresponds to the conditions in our growth media. In this respect, when considering encapsulated *K. marxianus* as a candidate microbe for the production of platform compounds, Figure 3A shows that in both cultures the amounts of total organic acids were practically unchanged from the levels that were introduced into the MRSLL growth medium. Therefore, this kind of fermentation might be useful for biotechnological production of various compounds from lactose in a multi-stage system, successively followed by separation processes (e.g., simultaneous production of acids and alcohols).

**Figure 4:**
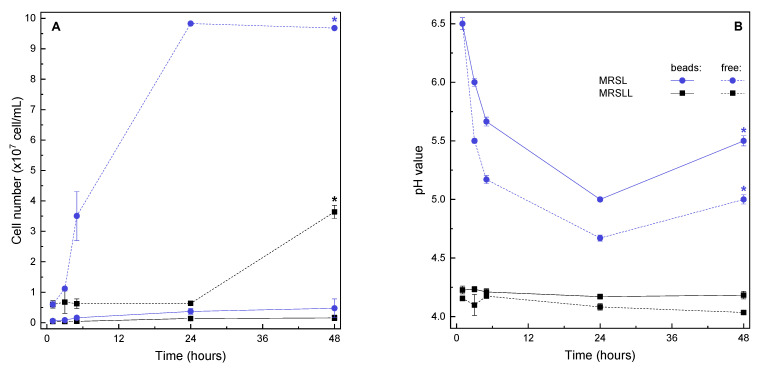
Incubation of *K. marxianus* in MRSLL and MRSL culture media (48 h, 28 °C) monitored by (**A**) cell proliferation and (**B**) environmental pH value. Data are means ±standard deviation (*n* = 3). Encapsulated culture in MRSLL was taken as the reference; dissimilar curves (*f*_2_ < 50) are marked with an asterisk (*).

In MRSLL, the initial pH value (4.2) was relatively well-maintained for broths containing free and encapsulated yeasts. The growth of the biomass in the liquid phase was slow and started to increase after the first day of incubation in the free culture. During hydrogel formation, some cells had set on the periphery of the microbeads (Figure 1), and in further handling steps, these cells might detach or even expel from the hydrogel due to, for example, the budding of yeasts. For this reason, it is important to measure cell concentration in the liquid phase even for encapsulated systems, as this will provide information on cell detachment. Based on the measured values, it is evident that some cells were detached throughout fermentation into MRSLL, albeit at very low concentrations with no apparent proliferation. (Figure 4).

Although cell growth was observed to be slow in all cases, sugar uptake and ethanol production peaked when negligible microbial growth was observed, providing opportunities for novel fermentation strategies in these conditions, including overall shorter fermentation periods, as a considerable decrease in time would not result in a considerable decrease in production, and multiple reuse of microbeads, as the shorter retention of microbeads within the individual batch would mean slower incremental growth of the yeasts within the bead, reaching overgrowth at a later time with a higher number of individual fermentations. Furthermore, as the growth of the yeasts continued after ethanol production had already stopped, we concluded that the produced ethanol amounts were not the main inhibitors, and that entrapping the yeast within the hydrogel network provided protection from unfavorable environmental factors. To examine this further, yeasts were incubated in MRSL (Figure 4B), where the initial pH was 6.5 and the final pH was lower for the free culture (5.0) than for the encapsulated culture (5.5). The higher pH change can be ascribed to higher levels of production in MRSL and to the absence of the pH buffering effect of lactate. An increase of pH values can be observed in MRSL after 24 h production, which can be attributed to CO_2_ equilibration and dissolution following the conclusion of fermentation. Furthermore, a typical growth curve was observed by yeasts suspended in MRSL, with the fastest growth rate in the first hours of fermentation and cessation of growth after 24 h, which is also in accordance with our preliminary observations (see Figure S1 in the Supplementary Materials) and other literature [55]. In MRSL, the amount of detached cells was lower when compared to suspended cells, but higher when compared to detached cells in MRSLL.

The discrepancy in growth within the yeast cultures that were freely suspended in MRSLL and MRSL indicates the inhibitory effect of lactic acid. Based on initial fluorescence microscopy measurements (Figure 1) and the observed lesser growth of the detached cells (Figure 4A vs. Figure 4B), this might indicate that upon detachment from the microbeads, the cells in MRSLL were introduced into a largely unhospitable environment that prevented their further overgrowth. Thus, encapsulation could provide a particularly valuable approach in such environments, as the uninhibited growth of detached cells, e.g., in enriched conditions, can cause significant problems. To prevent the release of cells at significant levels, additional encapsulation steps are often required, such as layering and coating, admixing lower cell concentrations with the carrier, etc. Furthermore, a possible explanation for the protective effect of alginate beads could be deduced from charge effects, as in MRSLL the lactic acid (pK_a_ of 3.86) is expected to be deprotonated. Likewise, the remaining carboxyl residues on alginate, which remain unbound by Ca^2+^ crosslinking, will exist almost entirely as anions. Thus, a deprotonated carrier could enable a more suitable carrier host environment and protection from a deprotonated inhibiting compound. However, further survival studies of yeast fermentation in hydrogel matrices and alginate-lactate microdiffusion analyses are required to fully understand these opportunities. Nevertheless, encapsulation systems based on carriers with the proposed inhibitor-expelling or/and -repelling properties would contribute greatly to design opportunities for novel cell delivery systems. This concept could be extended beyond charge-repulsion to other types of carrier exclusion (e.g., via varied hydrophobicity or steric hindrance) for extending various product inhibition metabolisms. Similarly, as the freely suspended cultures exhibited slower growth when compared to encapsulated yeasts, and as slower growth is more beneficial when biomass recycling is required, finely tuned carrier charge modulation might provide further design options for multiple cycles of microbead use.

## 3. Materials and Methods

### 3.1. Materials

Growth media components were from Merck (Darmstadt, Germany) and Sigma Aldrich (St. Louis, MO, USA). HPLC reagents and alginic acid (sodium salt) from brown algae (with 0.0175 Pa s viscosity at 1% [*w/w*] and 25 °C) were from Sigma Aldrich (St. Louis, MO, USA). Fluorescent stains were from ThermoFischer Scientific (Waltham, MA, USA). For the complete list of materials, see the Supplementary Materials.

### 3.2. Microorganisms and Initial Culture Media

Two types of lactic acid bacteria with demonstrated probiotic attributes [6] were used, i.e., *Lactiplantibacillus plantarum* of plant origin (ATCC 8014), obtained from Deutsche Sammlung von Mikroorganismen und Zellkulturen (Braunschweig, Germany) and *Lacticaseibacillus rhamnosus* GG of human origin (ATCC 53103) obtained from Valio (Helsinki, Finland). These cultures were incubated in de Man, Rogosa and Sharpe (MRS) broth by shaking (250 rpm) at 37 °C for 16 h, reaching an approximate optical density (A_600_) of 1.8 (corresponding to late exponential growth and estimated cell concentration at ~10^9^ CFU/mL [57,58]). The bacteria were harvested by centrifugation at 5000× *g* for 10 min at 24 °C and washed twice in sterile 0.9% NaCl prior to encapsulation.

We also used the yeast *Kluyveromyces marxianus* isolated from kefir grains (ZIM 1868) obtained from the Slovenian Collection of Industrial Microorganisms (registration code WDCM810, Ljubljana, Slovenia), which is a frequent companion to lactic acid bacteria in mixed cultures in dairy processing. The lactose-fermenting yeast culture was grown in modified MRS, where glucose was substituted with either lactose (denoted as MRSL) or lactose and lactic acid (denoted as MRSLL). See the Supplementary Materials (Table S1) for the exact media composition. In MRSL broth, the yeast culture was maintained by shaking (100 rpm) at 28 °C for up to 10 h, reaching an approximate optical density (A_650_) of 1.8–1.9, corresponding to late exponential growth and estimated cell concentration at ~10^8^ cells/mL). The measured growth curve of *K. marxianus* in MRSL is available in the Supplementary Materials (Figure S1). For encapsulation, the propagated yeast culture was decanted and transferred to the encapsulation carrier.

### 3.3. Encapsulation

Alginate microbeads were prepared with a vibrating nozzle encapsulator (B-395 Pro; Büchi Labortechnik, Flawil, Switzerland) equipped with a 2 L reaction vessel and a 150 μm nozzle. Aseptic conditions were maintained by autoclaving the reaction vessel prior to each encapsulation and aseptic handling of microorganisms and other materials. Microbial cultures were provided either as washed in saline solution or decanted in the corresponding culture medium and mixed with the carrier to achieve 2% alginate [*w/w*]. Working conditions of the device were tuned to each feed. The mixture with ~10^8^ cell/mL lactic acid bacteria was prepared at 1000 Hz, 1000 V, and a 3 mL/min feed rate. The mixture with ~10^7^ cell/mL yeasts was prepared at 1100 Hz, 1000 V, and a 2.5 mL/min feed rate (see Table S2 and Figure S2 in the Supplementary Materials for details on the chosen conditions and microbead appearance). The resulting microbeads were hardened for 30 min in 1.5% CaCl_2_ [*w/w*] with gentle agitation (250 rpm), then decanted and aseptically transferred by pipetting.

### 3.4. Survival of Free and Encapsulated Strains at Low pH

Tolerance to various environmental pH values (2.0, 5.0, and 7.0) for free and encapsulated probiotics (*L. plantarum* and *L. rhamnosus*) was examined in a relatively short-term window (up to 180 min) as a means of obtaining information of probiotic survival during gastric passage. Freshly prepared microcapsules (0.5 mL) or 0.25 mL (9 log cfu/mL) of free cell suspensions were introduced in 10 mL of appropriate buffer solutions (consisting of KH_2_PO_4_, NaH_2_PO_4_, NaOH, and H_3_PO_4_) and incubated at 37 °C and mixed (250 rpm).

Tolerance and activity to low environmental pH values in the presence of inhibiting metabolites (lactic acid) for free and encapsulated yeasts (*K. marxianus*) were examined throughout a 48 h fermentation period. The set pH value (4.2) was selected based on the expected amount of lactic acid at the point of completed milk fermentation by lactic acid bacteria in mixed-culture milk products (pH 4.2–4.6), such as kefir and ayran [59,60]. Freshly prepared microcapsules (2 mL), prepared by mixing 1 mL cell suspensions with 1 mL of 4% alginate) or 1 mL (8 log cfu/mL) of free cell suspensions, were introduced in 40 mL of MRSLL, sealed with an airlock and incubated at 28 °C.

### 3.5. Total Viable Cell Counts

When required, hydrogel dissolution was performed to release the entrapped microorganisms by mixing 1 mL microbeads with a 9 mL 10% sodium citrate solution followed by vortexing. Total microbial cell counts were determined by the drop plate method. For this process, 10 µL diluted inocula (10^−4^–10^−7^) were plated on appropriate agar plates and counted as colony-forming units (CFU) after a 24 h incubation period under anaerobic conditions at 37 °C.

### 3.6. Microscopy

Hydrogel bead morphology and particle size were investigated with a DM750 optical microscope equipped with a ICC50 digital camera (both from Leica, Wetzlar, Germany). Particle size analysis was performed over 100 particles per sample, using the Fiji open-source image-processing package, ImageJ software [61], version 1.52b, and Origin Pro 2015 software, version b9.2.272.

Confocal laser scanning microscopy was performed to estimate the spatial distribution and viability of the microbes at different time points during bead preparation and incubation. Freely suspended or encapsulated microorganisms were observed with the LSM 800 inverted Axio Observer Z1 microscope equipped with the AxioCam MRm3 and operated with ZEN 2.3 software, file version 2.3.16103.6401 (all equipment from Zeiss, Oberkochen, Germany). The samples were stained with SYTO 9 and propidium iodide fluorescent dyes (1:500 final stain dilution) and placed (20 μL) directly on 1.5# 20 × 60 mm coverslips, which were left uncovered to preserve the shape of the microbeads. In the confocal laser scanning mode, the optical slicing was performed in 3 μm steps by using a 20× objective. SYTO 9 and propidium iodide fluorescence was excited, respectively, with 488 nm (blue) and 561 nm (yellow) diode lasers. The images were visualized, pseudo-colored, and analyzed by ImageJ software [61].

### 3.7. HPLC

Fermentation of lactose by *K. marxianus* in the presence of lactic acid was monitored with high-performance liquid chromatography (Infinity 1260 system; Agilent Technologies, Santa Clara, CA, USA), equipped with an Aminex HPX-87H Column (300 × 7.8 mm, 9 µm particle size, from BioRad, Hercules, CA, USA). Isocratic elution was performed over 35 min at a flow rate of 0.6 mL/min, with a 5 mM sulfuric acid mobile phase and a 20 µL injection volume. The analysis was performed at 50 °C (T_autosampler_ = 10 °C, T_injector_ = 15 °C, T_detector_ = 29 °C) and the eluted components were determined with a refractive index detector. See the Supplementary Materials for information on the standards and calibration. All of the analyses were performed in triplicate.

### 3.8. Statistical Analysis

Student t-tests were performed to differentiate between the means with a 95% confidence interval (*p* < 0.05). For comparing production and growth curves, the similarity factor (f_2_) was calculated according to the guidelines of the Food and Drug Administration [62]. See the Supplementary Materials for details. Calculations were performed with the OriginPro 2018 SR1 b9.5.1.195 (OriginLab, Northampton, MA, USA) software package.

## 4. Conclusions

Electrostatic manufacturing allowed for the preparation of well-defined alginate microbeads (180–260 µm diameter), high cell-entrapment (95%) and viability (90%), and uniform distribution of encapsulated cells throughout the hydrogel matrix.

In short-term survival studies, results pointed to the varied inherent tolerances of *L. plantarum* and *L. rhamnosus* to isotonic starvation at low environmental pH values. Freely suspended and entrapped *L. plantarum* maintained relatively high viabilities at pH 2.0; after 180 min, survival was maintained in the encapsulated culture (19% improvement compared to the free culture). On the other hand, *L. rhamnosus* appeared to be less robust, as in the same conditions a prolonged response to the hardening solution was initially observed, and most of cells (free or entrapped) were shown to be dead within 60 min. The overall highest survival of *L. rhamnosus* was achieved in alginate microbeads at pH 7.0.

The native lactose-fermenting yeast *K. marxianus*, which is a frequent companion to lactic acid bacteria in mixed cultures derived from various food sources, showed prospective application of encapsulation. Encapsulated yeasts exhibited double product yields in lactose- and lactic acid-modified MRS growth media (compared to freely suspended cells, allowing further process optimization). In conjunction with the pH-responsive features of alginate, fermentation products, and cell growth, the results indicate that yeast cells were protected from inhibition by organic acids within the alginate hydrogel.

The tested system requires relatively straightforward production and has exhibited prospective possibilities for *L. plantarum* delivery in probiotics, as well as fermented food production with *K. marxianus*, especially in two-stage fermentation by mixed cultures. Subsequently, the yeasts could be added to the already partially acidified fermentation matrix (e.g., for fermented dairy products). Novel possible carrier design features arising from the observed results include the modification of surface charge properties for inhibitor expulsion and achieving balanced inhibitor penetration for the maintenance of high rates of production at lower growth rates of the encapsulated cells.

## Data Availability

The data presented in this study are available on request from the corresponding author.

## Supplementary Materials

Table S1: Composition of culture media (g/L) used for the cultivation of lactic acid bacteria and lactose-fermenting yeasts. Figure S1: Growth curve of *Kluyveromyces marxianus* in MRSL medium, incubated at 28 °C, with shaking (250 rpm). Table S2: Encapsulator working conditions and feed parameter screening. Nozzle diameter (150 μm) and feed rate (2.5 mL/min) were not changed. Shaded area denote chosen conditions. Figure S2: Increasing concentration of *K. marxianus* (from left to right; 0, 0.75, 1.875, 3.75 × 10^7^ cell/mL) in alginate microbeads, prepared with 2% alginate, 1.5% CaCl_2_, 1000 V, 1100 Hz, 150 μm nozzle and 2.5 mL/min feed rate. Figure S3: To determine possible microbial contamination, pure alginate hydrogel microspheres were prepared (shown on left, confocal micrograph) and stained with propidium iodide and SYTO 9 (shown on right, combined live/dead fluorescence micrograph), where a small amount of residual algal biomass (from the alginate extraction process) was present (hardly observable fluorescence on right picture). Furthermore, dissolved beads were checked for contamination with standard plating methods, where after 24 h incubation at 37 °C no CFU were detected. Figure S4: Visual appearance of alginate microbeads without (left column) or with (right column) lactic acid bacteria immediately upon addition of 10% sodium citrate (top row) or after vortexing (bottom row). Figure S5: Survival and spatial distribution of freely suspended (LP) *Lactiplantibacillus plantarum*, (LR) *Lacticaseibacillus rhamnosus* and (KM) *K. marxianus*. Top row shows differential interference contrast micrographs and bottom row shows cells stained by live (SYTO 9 dye)/dead (propidium iodide dye) kit. The red and yellow cells are dead cells, whereas only green cells are alive. Scale bar corresponds to 20 μm.
