# Supplementary material for: Polysaccharide Hydrogels for the Protection of Dairy-Related Microorganisms in Adverse Environmental Conditions
Source: Molecules. 2021 Dec 10;26(24):7484. doi: 10.3390/molecules26247484 (PMC8704654; doi:10.3390/molecules26247484)
Supplement: Supplementary file 1 [file molecules-26-07484-s001.zip › molecules-1482398-supplementary.pdf]

Polysaccharide hydrogels for the protection of dairy–related microorganisms in adverse environmental conditions

Supplementary Materials

Ilja Gasan OSOJNIK ČRNIVEC [1], Tigran NERESYAN [2], Yuliana GATINA [2], Vid KOLMANIČ BUČAR [1], Mihaela SKRT [1], Iztok DOGŠA [1], Bojana BOGOVIČ MATIJAŠIĆ [1], Irina KULIKOVA [2], Aleksei LODYGIN [2] and Nataša POKLAR ULRIH [1,3,*]

[1] Biotechnical Faculty, University of Ljubljana, Jamnikarjeva 101, SI-1000 Ljubljana, Slovenia

[2] Food Engineering and Biotechnology Faculty, North-Caucasus Federal University, Pushkin Street 1, Stavropol 355017, Russia

[3] The Centre of Excellence for Integrated Approaches in Chemistry and Biology of Proteins (CipKeBiP), Jamova 39, SI-1000, Ljubljana, Slovenia

[*] Correspondence: author (natasa.poklar@bf.uni-lj.si)

1. Materials

Glucose, lactose and lactic acid (all HPLC grade), hydrochloric acid (≥37%), sulfuric acid (96%), calcium chloride, peptone from casein, meat extract, yeast extract, polyoxyethylene sorbitan monooleate (Tween 80), sodium alginate from brown algae (with 0.0175 Pa s viscosity at 1% [*w/w*] and 25 °C) were from Sigma Aldrich (USA). Triammonium citrate and sodium acetate were from Merck (Germany). Monopotassium phosphate (KH_2PO_4), dipotassium phosphate (K_2HPO_4), monosodium phosphate (NaH_2PO_4), sodium hydroxide (NaOH), phosphoric acid (H_3PO_4), magnesium sulfate ($MgSO_4$), manganese (II) sulfate ($MnSO_4$) and sodium azide (NaN_3), all p.a., were from Kemika (Croatia). Sodium chloride (NaCl) was from Honeywell Fluka (Germany). Propidium iodide and SYTO 9 fluorescent stains were obtained as a LIVE/DEAT™ Bacterial Viability Kit from ThermoFischer Scientific (USA). Ultrapure water (18.2 MΩ cm, 25 °C) was used for all aqueous solutions and all other requirements.

2. Microorganisms and culture media

Table S1: Composition of culture media (g/L) used for the cultivation of lactic acid bacteria and lactose fermenting yeasts.

Component	MRS [a]	MRSL	MRSLL
Peptone	10	10	10
Meat extract	10	10	10
Yeast extract	5	5	5
K_2HPO_4	2	2	2
Triammonium citrate ($C_6H_{17}N_3O_7$) [b]	2	2	2
Sodium acetate ($C_2H_3NaO_2$)	5	5	5
$MgSO_4 \times 7\ H_2O$	0.2	0.2	0.2
$MnSO_4 \times 4\ H_2O$	0.05	0.05	0.05
Tween 80	1	1	1
Glucose ($C_6H_{12}O_6$)	20	0	0
Lactose ($C_{12}H_{24}O_{12}$)	0	20	10
Lactic acid ($C_3H_6O_3$)	0	0	10

[a] (de Man, Rogosa, and Sharpe 1960), [b] triammonium citrate was omitted for encapsulation, to avoid possible dissolution of polysaccharide hydrogels.

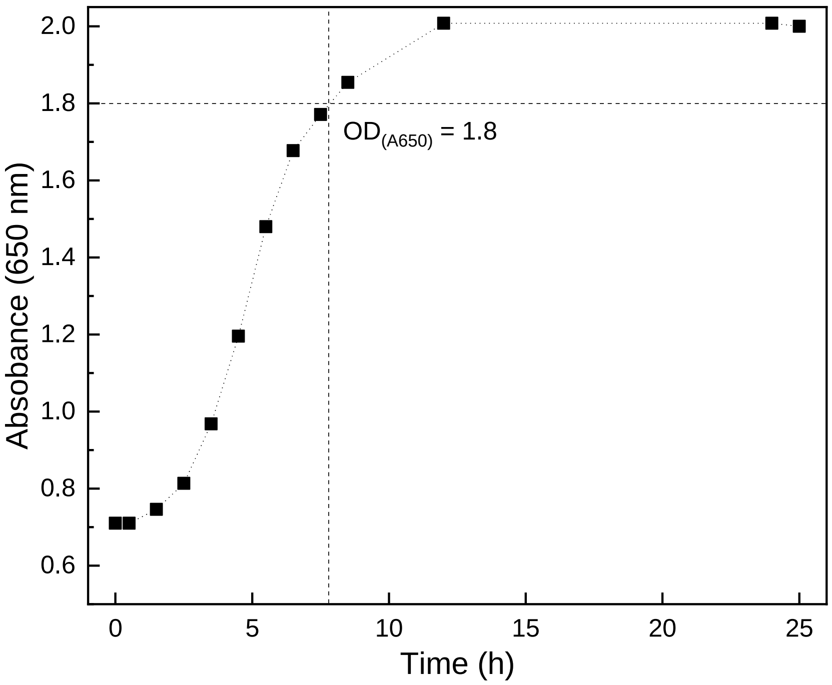

Figure S1: Growth curve of *Kluyveromyces marxianus* in MRSL medium, incubated at 28 °C, with shaking (250 rpm).

3. Encapsulation

Table S2: Encapsulator working conditions and feed parameter screening. Nozzle diameter (150 µm) and feed rate (2.5 mL/min) were not changed. Shaded area denote chosen conditions.

Feed and gelation components				Working conditions		Particle	
**Alginate (%)**	***K. marxianus* (cells/mL)**	**$CaCl_2$ (%)**	**Autoclaved (Y/N)**	**Voltage (V)**	**Frequency (Hz)**	**Shape (S/D)**	**Diameter (µm)**
2.0	-	1.5	N	1200	1100	S	169.5 ± 6.14^{abc}
2.0	-	1.5	N	1100	1100	S	177.2 ± 5.50^{defgh}
2.0	-	1.5	N	1000	1100	S	256.5 ± 41.7^{i}
2.0	-	1.25	N	1000	1100	S	177.1 ± 5.71^{defgh}
2.0	-	1.0	N	1000	1100	S	175.1 ± 13.6^{efgh}
1.5	-	1.5	N	1000	1100	S	176.6 ± 17.1^{defgh}
1.5	-	1.25	N	1000	1100	S	171.8 ± 8.41^{bchj}
1.5	-	1.0	N	1000	1100	S	173.0 ± 4.34^{bcehj}
1.0	-	1.5	N	1000	1100	D	146.7 ± 8.49^{k}
1.0	-	1.25	N	1000	1100	D	149.3 ± 6.70^{l}
1.0	-	1.0	N	1000	1100	D	157.1 ± 7.90^{mn}
2.0	-	1.5	N	1000	1100	S	256.5 ± 41.7^{i}
2.0	-	1.5	Y	1000	1100	S	177.9 ± 21.3^{dfgh}
2.0	3.75×10^7	1.5	Y	1000	1100	S	189.2 ± 22.1^{o}
2.0	1.875×10^7	1.5	Y	1000	1100	S	181.9 ± 15.7^{cefgh}
2.0	0.75×10^7	1.5	Y	1000	1100	S	175.0 ± 18.1^{gp}
1.5	-	1.5	N	1000	1100	S	176.6 ± 17.1^{defgh}
1.5	-	1.5	Y	1000	1100	S	169.6 ± 21.2^{abc}
1.5	3.75×10^7	1.5	Y	1000	1100	D	170.6 ± 22.7^{abcj}
1.5	1.875×10^7	1.5	Y	1000	1100	D	165.0 ± 16.9^{r}
1.5	0.75×10^7	1.5	Y	1000	1100	S	154.3 ± 17.7^{s}

Y – yes, N – no, S - spherical particles, D - deformed particles. Data are means ±standard deviation (n = 100). Letters (a–s) indicate significant differences ($P < 0.05$) between means.

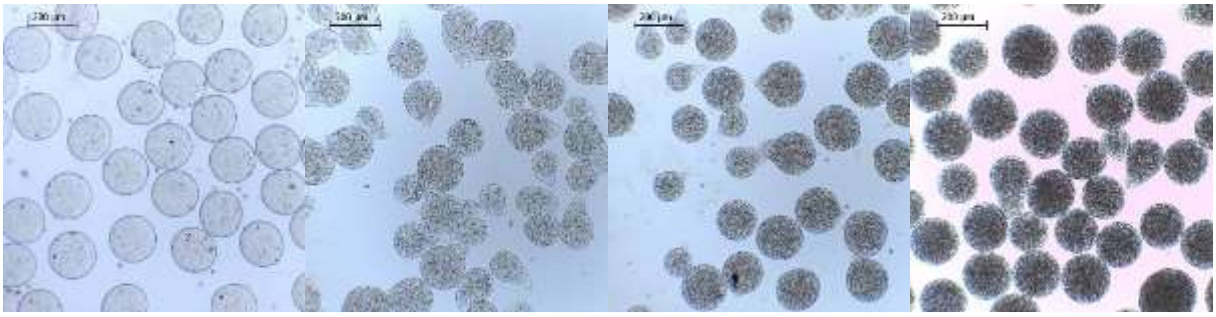

Figure S2: Increasing concentration of *K. marxianus* (from left to right; 0, 0.75, 1.875, 3.75 $\times 10^7$ cell/mL) in alginate microbeads, prepared with 2 % alginate, 1.5 % $CaCl_2$, 1000 V, 1100 Hz, 150 µm nozzle and 2.5 mL/min feed rate.

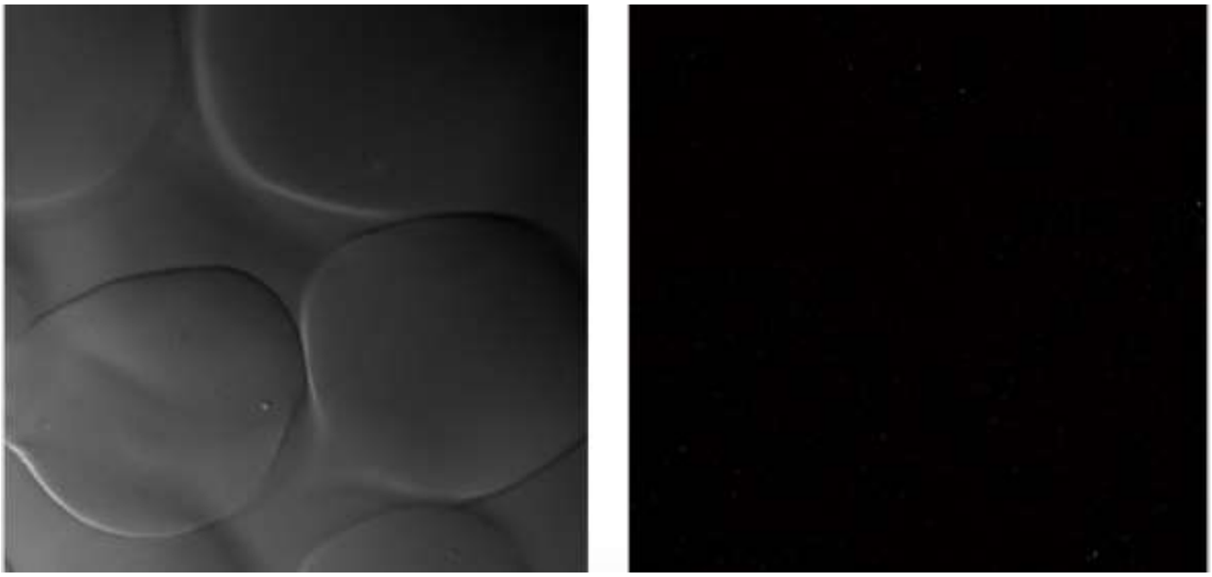

Figure S3: To determine possible microbial contamination, pure alginate hydrogel microspheres were prepared (shown on left, confocal micrograph) and stained with propidium iodide and SYTO 9 (shown on right, combined live/ dead fluorescence micrograph), where a small amount of residual algal biomass (from the alginate extraction process) was present (hardly observable fluorescence on right picture). Furthermore, dissolved beads were checked for contamination with standard plating methods, where after 24h incubation at 37 °C no CFU were detected.

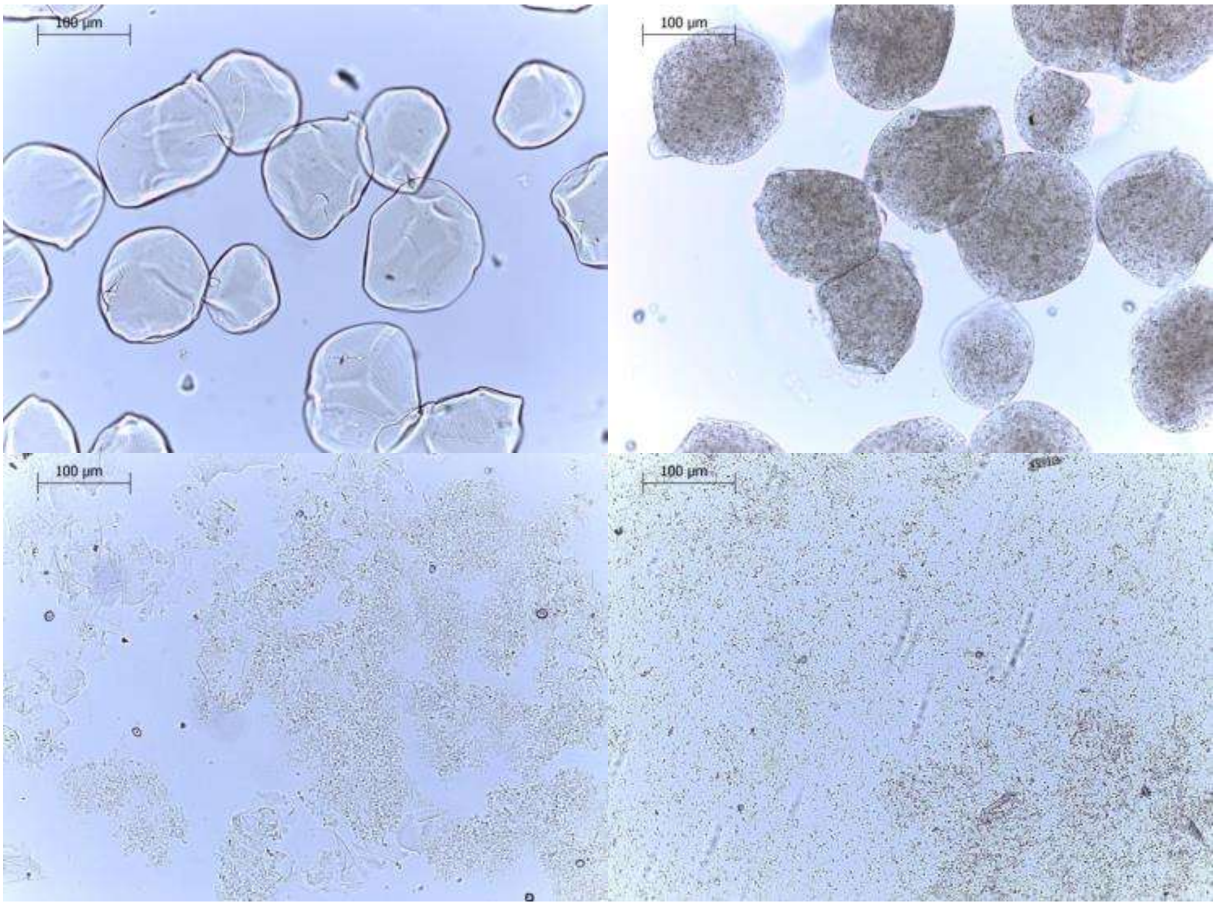

Figure S4: Visual appearance of alginate microbeads without (left column) or with (right column) lactic acid bacteria immediately upon addition of 10% sodium citrate (top row) or after vortexing (bottom row).

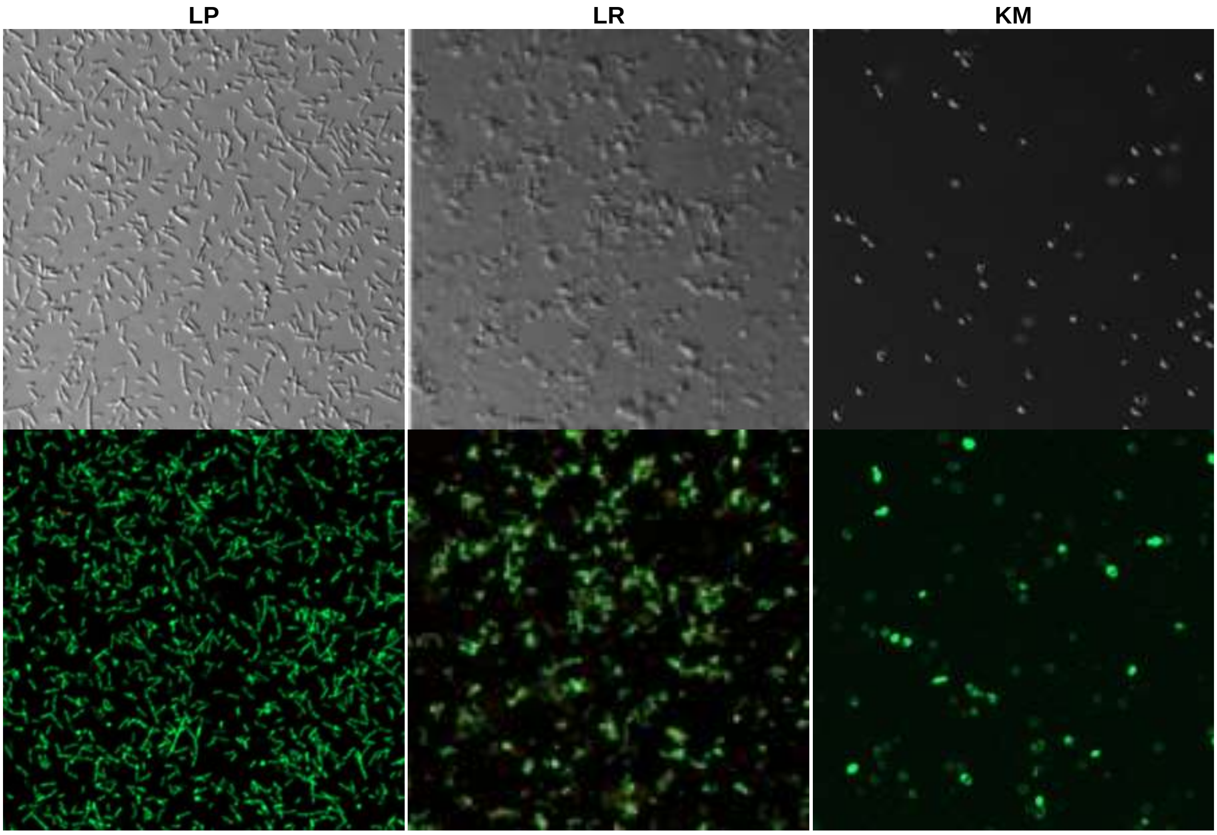

Figure S5: Survival and spatial distribution of freely suspended (LP) *Lactiplantibacillus plantarum*, (LR) *Lacticaseibacillus rhamnosus* and (KM) *K. marxianus*. Top row shows differential interference contrast micrographs and bottom row shows cells stained by live (SYTO 9 dye) / dead (propidium iodide dye) kit. The red and yellow cells are dead cells, whereas only green cells are alive. Scale bar corresponds to 20 µm.

4. High performance liquid chromatography

Fermentation of lactose by *K. marxianus* in the presence of lactic acid was monitored with high-performance liquid chromatography (Infinity 1260 system; Agilent Technologies, USA), equipped with an Aminex HPX-87H Column (300 × 7.8 mm, 9 µm particle size; from BioRad, USA). Isocratic elution was performed over 35 min at a flow rate of 0.6 mL/min, with a 5 mM sulfuric acid mobile phase and 20 µL injection volume. The analysis was performed at 50 °C ($T_{autosampler}$ = 10 °C, $T_{injector}$ = 15 °C, $T_{detector}$ = 29 °C) and the eluted were determined with a refractive index detector.

Linear regression for the 7-point lactose calibration curve was established in concentration range from 0.10 g/L to 10.0 g/L, with a linear regression correlation coefficient R^2 = 0,9999. The limit of detection and limit of quantification were calculated according to the guidelines of International Council for Harmonisation of Technical Requirements for Pharmaceuticals for Human Use, and were 0.02 g/L and 0.07 g/L, respectively.

Linear regression for the 8-point ethanol calibration curve was established in concentration range from 0.60 g/L to 10.0 g/L, with a linear regression correlation coefficient R^2 = 0,997. The limit of detection and limit of quantification were calculated according to the guidelines of International Council for Harmonisation of Technical Requirements for Pharmaceuticals for Human Use, and were 0.17 g/L and 0.52 g/L, respectively.

External standards of lactose (lactose monohydrate for biochemistry, 107660, Merck) and ethanol (absolute for analysis - EMSURE, 100983, Merck) were used for identification and quantification from the chromatograms obtained. All of the measurements were carried out in triplicate, and the data are expressed as means ±standard deviation (SD).

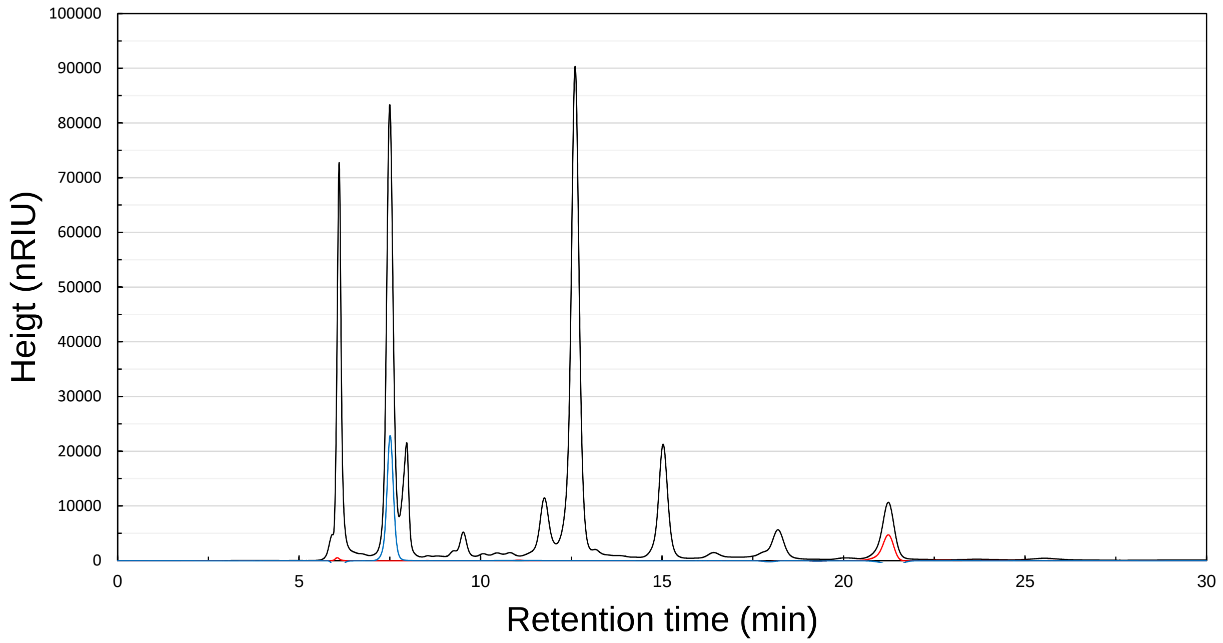

Figure S6: Representative HPLC chromatograms of the lactose (blue line) and ethanol (red line) standard in solution and MRSL broth sample (black line). The peaks at 7.5 min and 21.5 min corresponded to lactose and ethanol, respectively.

5. Statistical analysis

Student's t-tests were performed to differentiate between the means with a 95% confidence interval ($P < 0.05$). For comparing of the production and growth curves, the similarity factor (f_2) was calculated according to the guidelines of the Food and Drug Administration (Evaluation 4 (1997) 15–22), according to Equation (S1), where n = number of points, R_t = change in the reference compound at the given time point (in %), M_t = change in the monitored compound at the given time point (in %). Generally, f_2 values > 50 are considered to ensure equivalence of the two curves.

$$f_2 = 50\log\left\{100\times\left[1+\frac{1}{n}\sum_{i=1}^{n}\left(R_t - M_t\right)^2\right]^{-0.5}\right\} \tag{S1}$$

Analyses and calculations were performed with the OriginPro 2018 SR1 b9.5.1.195 (OriginLab, USA) software package.
